# Mechanistic and Evolutionary Insights from the Reciprocal Promiscuity of Two Pyridoxal Phosphate-dependent Enzymes*[Fn FN2]

**DOI:** 10.1074/jbc.M116.739557

**Published:** 2016-07-29

**Authors:** Valerie W. C. Soo, Yuliana Yosaatmadja, Christopher J. Squire, Wayne M. Patrick

**Affiliations:** From the ‡ Institute of Natural and Mathematical Sciences, Massey University, Auckland 0632,; the § School of Biological Sciences, University of Auckland, Auckland 1142, and; the ¶ Department of Biochemistry, University of Otago, Dunedin 9054, New Zealand

**Keywords:** directed evolution, enzyme mechanism, protein evolution, protein structure, pyridoxal phosphate

## Abstract

Enzymes that utilize the cofactor pyridoxal 5′-phosphate play essential roles in amino acid metabolism in all organisms. The cofactor is used by proteins that adopt at least five different folds, which raises questions about the evolutionary processes that might explain the observed distribution of functions among folds. In this study, we show that a representative of fold type III, the *Escherichia coli* alanine racemase (ALR), is a promiscuous cystathionine β-lyase (CBL). Furthermore, *E. coli* CBL (fold type I) is a promiscuous alanine racemase. A single round of error-prone PCR and selection yielded variant ALR(Y274F), which catalyzes cystathionine β-elimination with a near-native Michaelis constant (*K_m_* = 3.3 mm) but a poor turnover number (*k*_cat_ ≈10 h^−1^). In contrast, directed evolution also yielded CBL(P113S), which catalyzes l-alanine racemization with a poor *K_m_* (58 mm) but a high *k*_cat_ (22 s^−1^). The structures of both variants were solved in the presence and absence of the l-alanine analogue, (*R*)-1-aminoethylphosphonic acid. As expected, the ALR active site was enlarged by the Y274F substitution, allowing better access for cystathionine. More surprisingly, the favorable kinetic parameters of CBL(P113S) appear to result from optimizing the p*K_a_* of Tyr-111, which acts as the catalytic acid during l-alanine racemization. Our data emphasize the short mutational routes between the functions of pyridoxal 5′-phosphate-dependent enzymes, regardless of whether or not they share the same fold. Thus, they confound the prevailing model of enzyme evolution, which predicts that overlapping patterns of promiscuity result from sharing a common multifunctional ancestor.

## Introduction

Pyridoxal 5′-phosphate (PLP)^2^-dependent enzymes catalyze ∼4% of all the reactions that have been classified by the Enzyme Commission (EC) (1, 2). PLP-dependent enzymes are found in every organism, and it has been estimated that 1.0–1.5% of all genes in the genomes of free-living prokaryotes code for them (3).

Almost all known PLP-dependent enzymes catalyze reactions involving the Cα, Cβ, and Cγ atoms of amino acids or other metabolites with an amino group. Examples of the reactions catalyzed include racemization, elimination, transamination, decarboxylation, β- and γ-replacement, and retro aldol cleavage. Mechanistically, these reactions share the same first step, which is the formation of an external aldimine between the substrate and PLP (2, 4). The cofactor plays a catalytic role in labilizing one of the bonds at Cα of the substrate, lowering the activation barrier for forming a carbanionic intermediate. The roles of the protein moiety are largely to ensure cleavage of the correct bond at Cα and to guide the reaction of the intermediate toward the correct product (5).

Historically the PLP-dependent enzymes have been classified into five different fold types (6, 7), although this classification has recently been expanded to include two more folds that use PLP in a different mechanistic context (to stabilize radical intermediates) (2). Curiously, fold types I to V all use the same “phosphate-binding cup” to anchor PLP in the active site (8). We have argued that this is evidence for the ancient recruitment of a single cofactor-binding subdomain into the various extant folds (via non-homologous recombination) (9). This model implies partial homology between the fold types. However, for simplicity here we will refer to enzymes with different fold types as being non-homologous. Furthermore, the reaction specificities of PLP-dependent enzymes do not correlate strictly with their fold type. For example, aminotransferases that adopt fold types I and IV are known (2).

Together, these features raise two fundamental questions about the evolution of PLP-dependent enzymes. First, how have proteins with different folds evolved to control the reactivity of the cofactor? Second, what evolutionary processes or constraints explain the observed distribution of functions among folds?

In this study, we have addressed these questions by focusing on the two PLP-dependent enzymes alanine racemase (ALR) and cystathionine β-lyase (CBL). Previously, we showed that overexpression of ALR rescued an *Escherichia coli* strain in which the gene encoding CBL (*metC*) had been deleted (10). CBL is essential for *E. coli* to grow on minimal media, as it catalyzes the penultimate step in methionine biosynthesis. We were intrigued that ALR appeared to possess promiscuous CBL activity, as the two enzymes are not homologues (with the possible exception of their phosphate-binding cup subdomains). ALR (EC 5.1.1.1) and CBL (EC 4.4.1.8) are also unrelated with respect to the physiologically relevant substrates they recognize and the overall reactions they catalyze.

**FIGURE 1.**
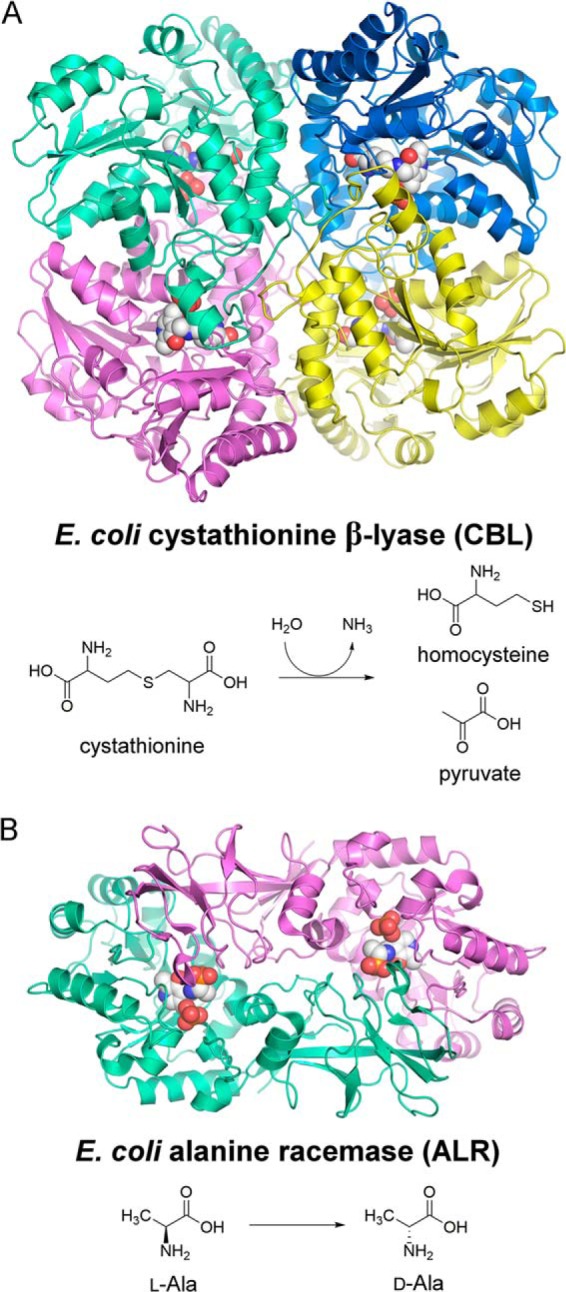
*A,* structure of the CBL tetramer (PDB entry 1CL1) (11) and the reaction it catalyzes in methionine biosynthesis. *B,* structure of the ALR dimer (PDB entry 2RJG) (15) and the reaction it catalyzes to provide d-alanine for peptidoglycan biosynthesis. In each structure, the locations of the active sites are highlighted by the PLP cofactor, which is shown in space-filling format.

*E. coli* CBL is a homotetramer that catalyzes the formation of l-homocysteine from l-cystathionine (Fig. 1*A*). Each monomer adopts the type I PLP-binding fold and includes the following three domains: the N-terminal domain contributes to tetramerization; the C-terminal domain consists of an anti-parallel β-sheet with α-helices on the solvent side; and the middle domain harbors PLP, which is found in a parallel seven-stranded β-sheet covered by adjacent β-helices (11). In the resting state, PLP forms an internal aldimine with active site residue Lys-210. The enzyme has been identified as a potential antibiotic target, as its inhibition impacts protein synthesis and also DNA synthesis (via the methyl donor, *S*-adenosylmethionine) in bacteria (12).

ALR interconverts l-Ala and d-Ala, with the latter being an essential constituent of the cell wall peptidoglycan. Indeed, the absence of d-Ala leads to a defective cell wall and cell death. The essentiality of ALR for cell wall biosynthesis renders it an attractive antibiotic target (13, 14). The enzyme is a homodimer, formed by head-to-tail association of two monomers. Each monomer adopts a type III PLP-binding fold (Fig. 1*B*). The N-terminal domain of each monomer is a (βα)_8_ barrel; the C-terminal domain mainly comprises β-strands (15, 16). The active site is found in the core of the (βα)_8_ barrel, where PLP forms an internal aldimine via a Schiff base linkage with Lys-34. An extensive and classic series of structural, mutagenic, and modeling analyses have established a two-base mechanism for ALR (16–22). In the *E. coli* enzyme, Tyr-255′ (with the prime indicating a residue from the opposing monomer than Lys-34) acts as the catalytic base that deprotonates l-Ala. Lys-34 acts as the catalytic acid, protonating the carbanionic intermediate to yield d-Ala. The roles of Tyr-255′ and Lys-34 are reversed during the racemization of d-Ala into l-Ala.

Here, we characterize the promiscuous CBL activity of ALR and show that it can be improved by directed evolution. We also demonstrate a novel example of reciprocal promiscuity in the absence of homology; CBL is a promiscuous and evolvable ALR. Thus, our study also highlights the short mutational routes that may have been sufficient to evolve families of homologous PLP-dependent enzymes with disparate reaction specificities.

## Results

### 

#### 

##### ALR Overexpression Rescued E. coli Lacking CBL Activity

*E. coli* Δ*metC* is unable to grow on M9/glucose agar; however, in our hands it formed colonies on medium supplemented with 0.1 mm l-methionine after 1–2 days of incubation. This demonstrated that deleting CBL ablates methionine biosynthesis, as expected. *E. coli* Δ*metC* that harbored a plasmid-encoded CBL (pCA24N-*metC*) survived in the absence of exogenous l-methionine (supplemental Table S1).

Previously, we showed that ALR overexpressed from a high copy plasmid (pCA24N-*alr*) could rescue *E. coli* Δ*metC*, with colony formation taking 5–6 days when expression was induced with 50 μm isopropyl β-d-1-thiogalactopyranoside (IPTG) (10). As with all of the open reading frames (ORFs) in the ASKA collection (23), the 3′ end of the *alr* insert is fused to the gene for green fluorescent protein (GFP) in pCA24N-*alr*. Therefore, we removed the GFP tag to confirm that complementation was due to ALR itself and was not a peculiarity of the ALR-GFP fusion. Cells overexpressing the GFP-less ALR did form colonies, after a shorter incubation period (3–4 days) (supplemental Table S1). Throughout this study, no growth was observed for the negative control (an *E. coli* Δ*metC* clone that harbored the empty vector without a gene insert, pCA24N-NoIns (24)).

This complementation assay appeared to show that ALR possessed promiscuous CBL activity. However, we also considered the possibility that ALR might rescue the methionine auxotrophy of *E. coli* Δ*metC* via a metabolic bypass, rather than by catalyzing the CBL reaction. To test this, we overexpressed ALR in an *E. coli* strain lacking the enzyme that follows CBL in the biosynthetic pathway, methionine synthase. The growth of this strain (*E. coli* Δ*metE*) was tested on M9/glucose medium. If ALR overexpression had led to the growth of this strain, then this would have suggested that the cell was (somehow) rerouting metabolic flux to synthesize methionine by a previously undescribed pathway. Examples of this phenomenon have been characterized for a different metabolic pathway (25). However, we observed no growth of *E. coli* Δ*metE* expressing ALR, in up to 1 week of incubation (supplemental Table S1). This demonstrated that ALR overexpression did not lead to methionine production via an alternative pathway.

Next, we attempted to measure the CBL activity of purified His_6_-tagged ALR by using 5,5′-dithiobis(2-nitrobenzoic acid) (DTNB) to detect the formation of homocysteine. However, no CBL activity was detected *in vitro*, at an enzyme concentration of 7 μm, concentrations of the substrate (l-cystathionine) up to 10 mm, and at temperatures of 25, 30, or 37 °C. We could not use higher l-cystathionine concentrations due to its low solubility (∼5.6 mg·ml^−1^ in 0.04 n HCl). In our control reactions that lacked enzyme, we observed the spontaneous decomposition of DTNB at an average background rate of ≤0.35 μm/min. This places an approximate upper limit on the CBL activity of ALR. In these assays we would have observed turnover of l-cystathionine if it had been greater than ∼0.05 per min per active site.

##### Directed Evolution to Improve the CBL Activity of ALR

Promiscuous activities are often orders of magnitude lower than native activities, and therefore, they can be exceedingly difficult to detect *in vitro* (25, 26). A simple strategy to circumvent this problem is to improve the promiscuous activity by directed evolution. The underlying assumption is that directed evolution approaches are unlikely to yield an activity that was not there before, although they are highly effective at improving weak but pre-existing functions (27).

We subjected the *alr* gene to a low mutation rate error-prone PCR (epPCR) and cloned the products into pCA24N. The resulting plasmids were used to transform *E. coli* Δ*metC*, yielding a library that contained 3.4 × 10^7^ *alr* variants. Sequencing the *alr* gene inserts (each 1,077 bp) from 20 colonies allowed us to use PEDEL-AA (28) to estimate the mean mutation rate at 0.8 base substitutions per allele, which corresponded to an average of 0.6 amino acid substitutions per ALR variant. The library was regrown and plated on M9/glucose with a reduced IPTG concentration (5 μm, instead of 50 μm) to increase the selection stringency. Under these conditions, the parental strain (*E. coli* Δ*metC* harboring pCA24N-*alr*) took 12–13 days to form colonies. Clones with higher CBL activity were selected based on their ability to form colonies more rapidly than the parental strain.

After only 2 days of incubation, we picked 32 colonies for further analysis. The growth phenotypes conferred by the selected *alr* variants were verified by isolating plasmid DNA from each colony and reintroducing it into fresh aliquots of *E. coli* Δ*metC*. Sequencing revealed that a single genotype, encoding the ALR(Y274F) mutant, dominated the pool with a frequency of 25/32 = 78% (Table 1). All of the other selected clones also possessed the Y274F mutation, in addition to 1–2 substitutions at other positions. The ubiquity of the Y274F mutation provided strong evidence that it was essential for improving the CBL activity of ALR.

**TABLE 1:** **ALR mutants selected from an epPCR library for their ability to rescue *E. coli* Δ*metC***

Variant	Frequency	Mutation(s)
ALR-1	25	Y274F
ALR-2	1	Y274F, A335T
ALR-3	1	R266S, Y274F
ALR-4	1	M29I, Y274F
ALR-5	1	T97S, P252R, Y274F
ALR-6	1	I242V, Y274F
ALR-7	1	L241M, R263H, Y274F
ALR-8	1	V150I, Y274F

Of the selected mutations, Y274F is the only substitution located in the vicinity of the active site. Tyr-274′ is found in the innermost part of the substrate entranceway (15), and it is conserved between bacterial alanine racemases (29, 30). The other nine mutated residues (Table 1) are either found on the surface of the dimeric enzyme or, in the cases of the buried residues Thr-97 and Leu-241, they appear to play simple structural roles on β-strands, where they have been conservatively replaced with serine and methionine in variants ALR-5 and ALR-7, respectively. Nevertheless, we were wary that distal residues can play important functional roles. Therefore, we used the Partial Order Optimum Likelihood (POOL) server (31) to predict and rank the functionally important residues in ALR, using PDB entry 2RJG as the input. For any given structure, all of the functionally important residues are typically in the top 8–10% of those ranked by POOL (31). The two Tyr-274 residues in the ALR dimer had the 11th and 16th highest POOL scores of 716 total residues (*i.e.* inside the top 2.5%). All of the other mutated residues in Table 1 had POOL scores outside of the top 35%. Thus, the additional substitutions in variants ALR-2 to ALR-8 were likely to be neutral or mildly deleterious, and we focused our attention on ALR(Y274F).

##### Kinetic Characterization of ALR(Y274F)

We purified His_6_-tagged ALR(Y274F) and assayed it for cystathionine β-lyase activity, which was found to be low but measurable (Table 2). At 0.85 s^−1^·m^−1^, the *k*_cat_/*K_m_* was ∼2.7 × 10^6^-fold lower than that of CBL for the same reaction. This was mainly due to a poor *k*_cat_ of ∼10 turnovers/h/active site. In comparison, the *K_m_* value of ALR(Y274F) for l-cystathionine was only 85-fold lower than that of CBL for its native substrate, and at 3.3 mm, it was comparable with that of wild type ALR for l-Ala (*K_m_* = 0.87 mm).

**TABLE 2:** **Steady state kinetic parameters of ALR, CBL, and their evolved variants** All values are the mean ± S.E. for three independent experiments (each including duplicate assays), except for the ALR(Y274F) assays with cystathionine, in which *n* = 6. The *k*_cat_ values are reported per active site.

Enzyme	Cystathionine → homocysteine + Pyruvate + NH_3_			l-Ala → d-Ala		
	*k*_cat_	*K_m_*	*k*_cat_/*K_m_*	*k*_cat_	*K_m_*	*k*_cat_/*K_m_*
	*s*^−*1*^	*mm*	*s*^−*1*^·*m*^−*1*^	*s*^−*1*^	*mm*	*s*^−*1*^·*m*^−*1*^
ALR	ND*^a^*	ND*^a^*	<0.85	55 ± 4	0.87 ± 0.15	6.4 × 10^4^
ALR(Y274F)	(2.8 ± 0.3) × 10^−3^	3.3 ± 1.0	0.85	40 ± 5	1.5 ± 0.6	2.6 × 10^4^
CBL	90 ± 14	(3.9 ± 0.7) × 10^−2^	2.3 × 10^6^	3.3 ± 0.6	51 ± 4	65
CBL(P113S)	95 ± 3	0.12 ± 0.02	7.9 × 10^5^	21.6 ± 0.4	58 ± 3	3.7 × 10^2^
CBL(P113A)	81 ± 11	0.31 ± 0.07	2.6 × 10^5^	4.6 ± 0.5	23 ± 5	2.0 × 10^2^
CBL(P113T)	30 ± 2	0.21 ± 0.02	1.4 × 10^5^	5.8 ± 0.5	39 ± 4	1.5 × 10^2^
CBL(P113Q)	96 ± 8	0.12 ± 0.02	8.0 × 10^5^	3.6 ± 0.6	33 ± 10	1.1 × 10^2^
CBL(Y111F)	0.07 ± 0.01	(2.3 ± 1.9) × 10^−2^	3.1 × 10^3^	ND*^a^*	ND*^a^*	∼0.011

*^a^* ND means, not detected.

The promiscuous aminotransferase and aldolase activities of the ALR from *Geobacillus stearothermophilus* have been improved by site-directed mutagenesis (32, 33). In these examples, improvements in the new activities were accompanied by drastic reductions in the alanine racemase activity, by 3–4 orders of magnitude. In contrast, the improvement of CBL activity in ALR(Y274F) was accompanied by a weak trade-off in its native activity. *In vitro* alanine racemization assays showed that ALR(Y274F) had a 1.4-fold decrease in *k*_cat_ and a 1.7-fold increase in *K_m_*, compared with ALR (Table 2).

##### Structures of ALR(Y274F)

Given the location of Tyr-274′ at the entrance to the active site, it seemed likely that mutation to a smaller Phe residue was useful for allowing the new (and larger; Fig. 1) substrate, cystathionine, to bind productively. To confirm this hypothesis, we used x-ray crystallography to solve the structures of the His_6_-tagged ALR(Y274F) variant and an inhibitor-bound form. The inhibitor, (*R*)-1-aminoethylphosphonic acid (l-Ala-P), is a phosphonate analogue of l-Ala that is a substrate for the transaldimination reaction, in which the Schiff base linkage between Lys-34 and PLP in the active site of the resting enzyme is displaced by the amino group of the incoming substrate. Thus, l-Ala-P forms a stable external aldimine with PLP in the ALR active site (17).

**FIGURE 2.**
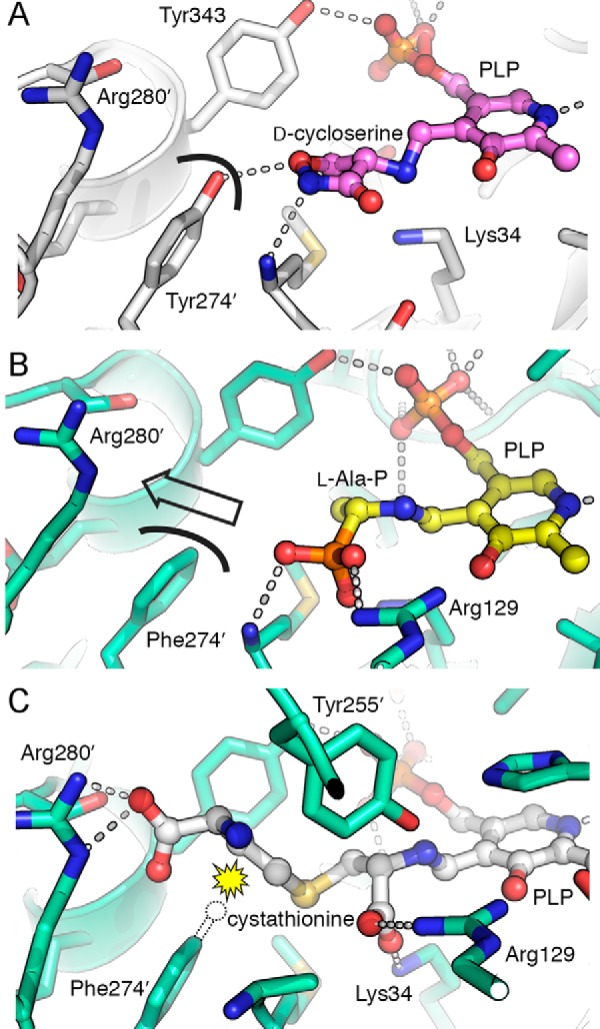
**Structural analysis of the Y274F mutation in ALR.** *A,* in the structure of wild type ALR inhibited by d-cycloserine (PDB entry 2RJH) (15), Tyr-274′ is positioned to form a hydrogen bond with the inhibitor. The *curved black line* shows the surface at the tyrosine hydroxyl group. *B,* active site of ALR(Y274F) with the l-Ala-P-PLP external aldimine, shown in the same orientation as *A*. The *curved black line* represents the surface of Phe-274′. The lack of a hydroxyl group opens a pathway from the substrate toward Arg-280′ (indicated with an *arrow*). *C,* modeled position of l-cystathionine in the ALR(Y274F) active site, docked using GOLD. The distal carboxylate of the substrate is perfectly positioned to interact with Arg-280′. A *dotted outline* indicates the position that the Tyr-274′ hydroxyl group occupies in the wild type ALR structure. The *yellow starburst* indicates the steric clash that would occur between this hydroxyl group and cystathionine, disfavoring substrate binding. The Y274F mutation removes this steric clash.

As expected, the Y274F mutation had no significant effect on the overall architecture of ALR. A pairwise comparison of the uninhibited ALR and ALR(Y274F) dimers (from PDB entries 2RJG and 4WR3, respectively) using PDBeFold (34) gave a root-mean-square deviation of only 0.26 Å over all 718 Cα atoms. In the wild type ALR structure, the hydroxyl group of Tyr-274′ forms a hydrogen bond (2.6 Å) with a crystallographic sulfate, and it has been proposed that this sulfate mimics the position of l-Ala on its route into the active site (15). When the enzyme was inhibited by d-cycloserine, the Tyr-274′ side chain hydroxyl formed a 3.2-Å hydrogen bond with the hydroxyimine moiety of the inhibitor instead (Fig. 2*A*). Although we also observed crystallographic sulfates in the structure of ALR(Y274F), there was neither a sulfate nor a water molecule in the position equivalent to that observed in the wild type structure. One implication is that removing the ability to form the hydrogen bond (by mutating Tyr-274 to Phe) has perturbed l-Ala binding; the increased *K_m_* for l-Ala in ALR(Y274F) supports this hypothesis.

Binding of l-Ala-P to ALR(Y274F) did not induce any significant conformational changes in the dimer (root-mean-square deviation = 0.16 Å over 715 Cα atoms), although we also note that the ligand-bound structure was generated by soaking. It is possible that the crystal lattice may have somewhat restricted conformational changes, although our results are consistent with those reported for the structure of the *G. stearothermophilus* ALR complexed with l-Ala-P, which was obtained by co-crystallization (17). Formation of the external aldimine tilts the plane of the PLP pyridine ring by ∼10° toward Tyr-255′. This has been observed previously (15, 17, 21). Tyr-255′ is positioned to act as the catalytic base, with a distance of 3.3 Å from its side chain hydroxyl group to Cα of the substrate. The side chain amino group of the catalytic acid, Lys-34, is 4.1 Å from Cα. As described previously (17), there was clear electron density for the l-isomer but not the d-isomer of the inhibitor, confirming that there had been no observable racemization of l-Ala-P.

The orientation of l-Ala-P in the active site of our ALR structure illuminates the role of the Y274F mutation in enhancing CBL activity. Removing the side chain hydroxyl of Tyr-274′ opens a pathway toward Arg-280′, without otherwise disturbing the structure of the active site (Fig. 2*B*). We hypothesized that cystathionine could be accommodated in the enlarged pocket formed by the Y274F mutation. To test this hypothesis, a cystathionine-PLP adduct was built and docked into the active site of ALR(Y274F) using GOLD. A scaffold matching constraint on the l-Ala-P molecule was used in the docking protocol to fix the PLP moiety of cystathionine-PLP in place, while allowing cystathionine to rotate freely and assume a biologically relevant configuration. In this docked model, the Cα atoms of l-Ala-P and cystathionine overlay, and the distal carboxylate of cystathionine is perfectly positioned to engage in a salt bridge with the side chain of Arg-280′ (Fig. 2*C*). However, the presence of the wild type tyrosine at position 274′ partially occludes the space between PLP and Arg-280′ and therefore is predicted to result in a steric clash with cystathionine (Fig. 2*C*).

**FIGURE 3.**
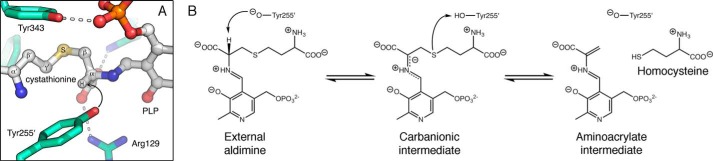
**Tyr-255′ is positioned to play a catalytic role in cystathionine β-elimination by ALR(Y274F).** *A,* when l-cystathionine-PLP is docked into the active site using GOLD, the side chain oxygen atom of Tyr-255′ is ∼2.6 Å from the proton that it is poised to abstract from the substrate (*black arrow*). *B,* tentative reaction scheme for ALR(Y274F)-catalyzed cystathionine β-elimination. Tyr-255′ has an unusually low p*K_a_* (20), so it is assumed to be in the phenolate form for proton abstraction (22). Unlike in CBL, the pyridine nitrogen is not protonated, because of the proximity of Arg-209 in the ALR active site. This results in a resonance-stabilized carbanionic intermediate of unusually high energy (45). By analogy with the CBL mechanism (35), we predict that Tyr-255′ reorients to protonate the leaving group (homocysteine). This would yield the PLP derivative of aminoacrylate, which is the substrate for reverse transaldimination (35), to form iminopropionate and to regenerate the Lys-34-PLP Schiff base. The nature of the carbanionic intermediate and inefficient proton transfer from Tyr-255′ to the leaving group are likely to explain the very poor turnover observed for ALR(Y274F).

As with almost all PLP-dependent enzymes, ALR and CBL each catalyze transaldimination to yield an external aldimine. Furthermore, the next step in the mechanism of each enzyme is to abstract the proton from Cα of the substrate (21, 22, 35, 36). Although an active site lysine is responsible for this step in CBL, Tyr-255′ plays the same role in ALR when the substrate is l-Ala (21). Given the shared stereochemistry of the native and promiscuous substrates (*i.e.* l-Ala and l-cystathionine), and the results of our docking (Fig. 2*C*), it is highly likely that Tyr-255′ deprotonates l-cystathionine once it has formed the external aldimine in the active site of ALR(Y274F) (Fig. 3). However, further mutagenesis will be required to test this hypothesis rigorously.

##### Search for Promiscuous Alanine Racemases

With our interest in PLP-mediated promiscuity piqued, we next set out to identify *E. coli* proteins with promiscuous alanine racemase activity. We rationalized that other racemases/epimerases, other (βα)_8_ barrel enzymes, or other PLP-dependent enzymes might be capable of catalyzing alanine racemization. In *E. coli*, deleting both alanine racemase genes (*alr* and *dadX*), leads to death unless d-Ala is supplied exogenously (37). Therefore, we conducted a genome-wide multicopy suppression study with *E. coli* strain MB2795 (Δ*alr* Δ*dadX*). After transforming *E. coli* MB2795 with the pool of ASKA plasmids, we spread ∼36,000 transformed cells on IPTG-supplemented LB agar, in the absence of d-Ala. After 4 days, nine colonies had formed. DNA sequencing revealed that these clones harbored four plasmid-encoded ORFs as follows: *alr* (four colonies), *dadX* (two colonies), *cycA* (two colonies), and *metC* (one colony). Retransformation tests confirmed that expressing each of these ORFs led to rescue of *E. coli* MB2795 and that their growth was not a result of cross-feeding between different clones on the same agar plate.

It was expected that IPTG-induced expression of either ALR or DadX would rescue the *E. coli* Δ*alr* Δ*dadX* strain. CycA is a serine/alanine/glycine:proton symporter, the expression of which presumably improved the ability of the cells to scavenge residual d-Ala from the selective medium. Most unexpectedly, expressing the *metC* product, CBL, also appeared to overcome the d-Ala auxotrophy of *E. coli* MB2795. The implication was that CBL could catalyze alanine racemization and therefore that ALR and CBL were reciprocally promiscuous. During the course of this study, another group also independently discovered the growth of revertants in the absence of d-Ala, when CBL was up-regulated in *E. coli* BL21(DE3) Δ*alr* Δ*dadX* (38). Increased CBL expression in the revertants was ascribed to point mutations in the *metJ* repressor. These results are consistent with our observation that the growth of *E. coli* MB2795 was effected by overexpression of plasmid-encoded CBL.

##### Improving the ALR Activity of CBL by Directed Evolution

We probed the evolvability of CBL using epPCR. The library consisted of 1.5 × 10^5^ mutated *metC* variants. Sequencing the *metC* gene inserts (each 1,185 bp) from 14 randomly chosen colonies revealed a mean mutation rate of 3.0 mutations per clone, corresponding to 2.0 amino acid substitutions per CBL variant (28). The cloned library was introduced into *E. coli* MB2795 and spread on LB agar with 5 μm IPTG. In the absence of exogenous d-Ala, the parental clone (*E. coli* MB2795 expressing His_6_-tagged CBL) took 5–7 days to form colonies under these conditions. In contrast, ∼4% of the library had formed colonies after incubation for 42 h. Previously, we observed some leaky expression from the T5-*lacO* promoter of pCA24N (26). Therefore, we retested 48 of the fast-growing clones on LB agar with no IPTG, and we identified 23 that formed colonies after 24 h of incubation.

DNA sequencing revealed that the population of 23 fast-growing clones was made up of 12 different variants (Table 3). Notably, all variants contained mutations at CBL residue Pro-113, immediately suggesting that this amino acid was a critical determinant of alanine racemization activity. Pro-113 was mutated to either serine, alanine, threonine, or glutamine. The most common mutation was P113S, which appeared in 17 of the 23 clones. This was followed by the P113A mutation, which was found in four clones. Single clones (CBL-6 and CBL-7) carried the P113T and P113Q mutations. Mutations were also identified at seven additional positions, in single variants (Table 3).

**TABLE 3:** **CBL mutants selected from an epPCR library for their ability to rescue *E. coli* MB2795 in the absence of IPTG**

Variant	Frequency	Mutation(s)
CBL-1	10	P113S
CBL-2	2	P113S; 1 silent
CBL-3	2	P113A
CBL-4	1	P113A, D247E
CBL-5	1	L34M, P113A; 1 silent
CBL-6	1	P113T
CBL-7	1	P113Q; 1 silent
CBL-8	1	P113S, A237V
CBL-9	1	P113S, N321S
CBL-10	1	P113S, A325V
CBL-11	1	P113S, F331T
CBL-12	1	P113S, A246V; 1 silent

None of the residues that were mutated in CBL are known to possess direct catalytic or substrate-binding roles. One residue (Leu-34, mutated to methionine in variant CBL-5) is located on the monomer-monomer interface of the N-terminal tetramerization domain (11). Four residues (Pro-113, Ala-237, Ala-246, and Asp-247) are in the middle domain of CBL, where the active site and PLP are located, but none contact the substrate or cofactor directly. The three remaining residues (Asn-321, Ala-325, and Phe-331) are in the C-terminal domain and are located on a solvent-exposed helix. Given the predominance of mutations at Pro-113, we surmised that the additional mutations were likely to be either neutral or mildly deleterious, with respect to the alanine racemization activity of CBL. Therefore, subsequent characterization focused on variants CBL-1, CBL-3, CBL-6, and CBL-7 (Table 3).

##### Kinetic Characterization of CBL Variants

We expressed and purified CBL and the four Pro-113 variants. Their l-alanine racemization activities were readily detected by using a coupled assay with d-amino acid oxidase (for converting the product, d-Ala, to pyruvate) and lactate dehydrogenase (for the NADH-linked reduction of pyruvate to lactate). We excluded the possibility that CBL might be acting as a deaminase (*i.e.* converting l-Ala into pyruvate), because a positive signal was only observed when d-amino acid oxidase was present. This confirmed that d-Ala was produced from l-Ala by CBL.

At 65 s^−1^·m^−1^, the *k*_cat_/*K_m_* of CBL for alanine racemization was almost 1,000-fold lower than ALR itself (Table 2). This was due to CBL possessing both a substantially larger *K_m_* for l-Ala (51 mm) and also a 17-fold lower *k*_cat_ (3.3 s^−1^). The evolved CBL variants had overall improvements in catalytic efficiencies that ranged from 1.7-fold for CBL(P113Q) to 5.7-fold for CBL(P113S). In the cases of the CBL P113A, P113T, and P113Q variants, these increases were mainly brought about by decreases in the *K_m_* value for l-Ala. In contrast, CBL(P113S) showed a 6.5-fold increase in *k*_cat_, whereas its *K_m_* value for l-Ala was similar to CBL (Table 2). Consistent with its predominance in the library selection experiment (Table 3), CBL(P113S) was the most active variant overall, with a *k*_cat_/*K_m_* that was only 170-fold lower than ALR.

The cystathionine β-elimination activities of the CBL Pro-113 variants were also assayed, to gauge the trade-off between the native and evolving activities. The variants showed cystathionine β-elimination activities that were reduced by 3–16-fold, compared with CBL itself (Table 2). CBL(P113T) was the only variant with a significantly reduced *k*_cat_; however, all four variants had increased *K_m_* values for l-cystathionine. In the P113A, P113T, and P113Q CBL variants, the mutations decreased cystathionine β-elimination activity by more than they increased alanine racemization activity. In contrast, CBL(P113S) showed a 2.9-fold reduction in cystathionine β-elimination, accompanied by a 5.7-fold increase in alanine racemization.

##### Toward a Functional Role for the Pro-113 Variants

**FIGURE 4.**
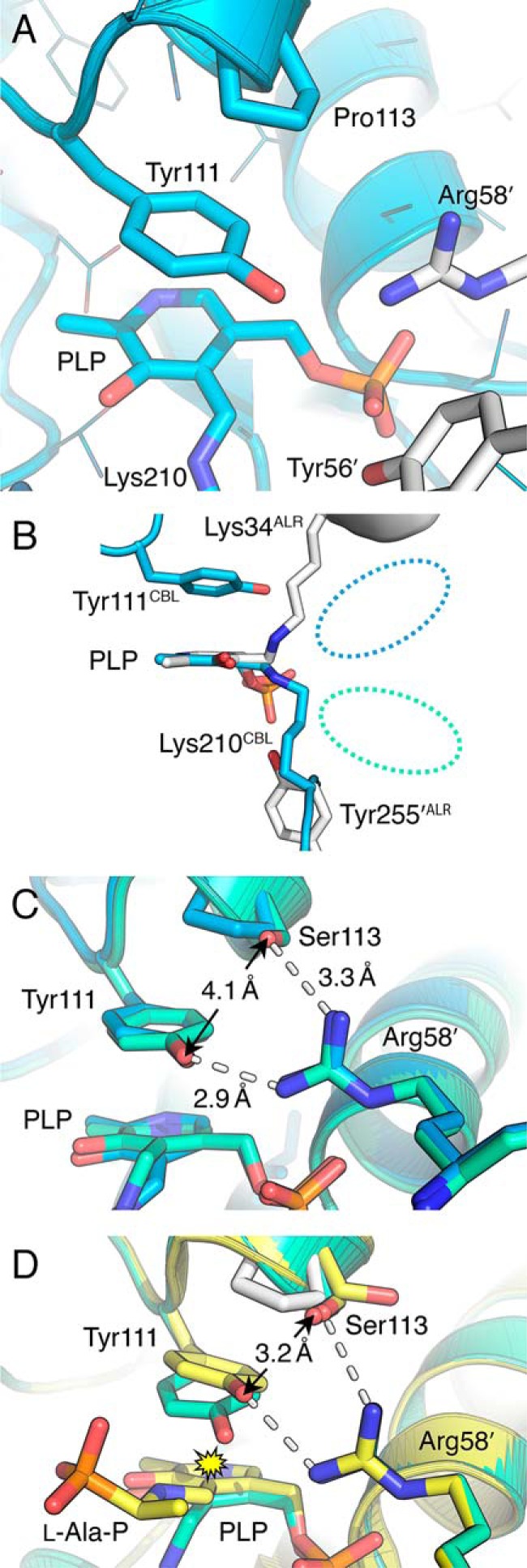
**Structural analysis of the P113S mutation in CBL.** *A,* in wild type CBL (PDB entry 1CL1) Pro-113 is positioned above Tyr-111, which in turn makes a stacking interaction with the pyridine ring of PLP (11). *B,* overlay of the catalytic residues in CBL (*blue*) and ALR (*white*), with the structures oriented so that their PLP cofactors superimpose. The approximate position occupied by incoming substrate is indicated with a *dashed blue oval* for CBL and a *dashed green oval* for ALR. *C,* CBL(P113S) (*green*) superimposed upon CBL (*blue*). Although there are no significant changes in the positions of active site residues, Arg-58′ is now within hydrogen bonding distance of Ser-113 in the mutant. *D,* overlay of the unliganded CBL(P113S) structure (*green*) and CBL(P113S) with an l-Ala-P-PLP external aldimine (*yellow*). Wild type residue Pro-113 is also shown in *white* to highlight the effect of the P113S substitution. Two rotamers of Ser-113 were observed in the inhibited structure; both are shown. The two hydrogen bonds involving Arg-58′ are the same length as in *C*. The *yellow starburst* highlights the steric clash that would occur if Tyr-111 did not pivot away from PLP in the inhibited structure.

Pro-113 is close to the active site of CBL, being located at the N-terminal end of helix 6 and packing directly above the active site residue Tyr-111 (Fig. 4*A*). Inspection of the wild type CBL structure (11) immediately suggested Tyr-111 and Lys-210 as candidates to act as the catalytic acid and base for alanine racemization by CBL, albeit positioned on the opposite faces of PLP than Tyr-255′ and Lys-34 in ALR (Fig. 4*B*). This orientation would mean that Lys-210 was responsible for deprotonating l-Ala (instead of Tyr-255′ in ALR) and that Tyr-111 was protonating the planar intermediate to yield d-Ala. We probed the role of Tyr-111 by constructing the site-directed mutant CBL(Y111F). A previous analysis of this variant showed that it was prone to inhibition by DTNB, while clarifying that Tyr-111 does not play a catalytic role in proton transfer during the CBL reaction (39). Consistent with these results, we observed a steep drop-off in *k*_cat_, but not *K_m_*, for CBL(Y111F) in our DTNB-coupled assays for cystathionine β-elimination (Table 2). Of more relevance here, and as predicted, CBL(Y111F) had ALR activity that was barely detectable above baseline. Although we could not saturate the enzyme, we estimated its *k*_cat_/*K_m_* to be ∼0.011 s^−1^·m^−1^, which is almost 6,000-fold lower than the ALR activity of wild type CBL (Table 2). This confirmed a critical catalytic role for Tyr-111 in alanine racemization and led us to hypothesize that the mutations at Pro-113 had been selected because they reoriented Tyr-111 to act more effectively as the catalytic acid in the conversion of l-Ala to d-Ala.

##### Structures of CBL(P113S)

To test our hypothesis, we solved the unliganded and l-Ala-P-bound structures of CBL(P113S). To our surprise, the mutation did not significantly affect the position of Tyr-111 in the unliganded structure (Fig. 4*C*). As observed in wild type CBL, the Tyr-111 side chain forms a π stacking interaction with the pyridine ring of PLP and a hydrogen bond (2.9 Å) with the side chain of Arg-58 from an adjacent monomer (*i.e.* Arg-58′). This hydrogen bond is the same length in the wild type structure (11). In CBL, Arg-58′ also makes a salt bridge with the phosphate group of PLP (11). All of these interactions remain in the CBL(P113S) structure. In the mutant, nitrogen atom NH1 of Arg-58′ is now within hydrogen bonding distance of the Ser-113 side chain (N-O distance of 3.3 Å; Fig. 4*C*).

We readily obtained a CBL(P113S) structure in which the ALR inhibitor, l-Ala-P, had entered the active site and formed a stable external aldimine. As with ALR(Y274F), this was obtained by soaking a pre-formed crystal with the inhibitor. Also, as we observed with ALR(Y274F), the inhibitor was in the l-isomer, and formation of the external aldimine was accompanied by a tilt of PLP by ∼10° away from Lys-210 (Fig. 4*D*). This induces a tilt in the Tyr-111 side chain by the same amount. These changes are smaller than those reported for the binding of a cystathionine analogue, l-aminoethoxyvinylglycine (AVG) to the wild type enzyme, which induced tilts of ∼30° in PLP and Tyr-111 (35). More substantially, the side chain also pivots by ∼25° along a perpendicular axis; therefore, the overall movement of Tyr-111 is best described as “up and away” from the l-Ala-P-PLP adduct (Fig. 4*D*). This repositioning of Tyr-111 avoids potential steric clashes between its hydroxyl group and C4′ of the cofactor (∼2.4 Å), as well as with Cβ of the l-Ala side chain (∼2.6 Å). By pivoting away, these “clash” distances increase to ∼3.8 and ∼3.6 Å, respectively (Fig. 4*D*).

The repositioning of Tyr-111 also brings its hydroxyl group closer to the side chain of Ser-113. In the inhibited structure, the electron density for Ser-113 is consistent with the side chain adopting two rotamers, with approximately equal occupancy (Fig. 4*D*). In one of these, the side chain is rotated toward bulk solvent. In the other, it occupies the same position as it does in the unliganded structure. In this latter conformation, the distance between the hydroxyl groups of Tyr-111 and Ser-113 decreases from 4.1 Å in the unliganded structure (Fig. 4*C*) to 3.2 Å when l-Ala-P is bound (Fig. 4*D*). Arg-58′ does not alter its position, and the 2.9-Å hydrogen bond with Tyr-111 remains intact.

We anticipated that the movement of Tyr-111 toward Ser-113 might alter the p*K_a_* of its hydroxyl group and therefore its ability to act as the catalytic acid during l-Ala racemization. The relevant side chain p*K_a_* values were estimated using PROPKA 3.1 (40). This analysis (Table 4) suggested that the introduction of the P113S mutation lowered the side chain p*K_a_* values of both Tyr-111 (Δp*K_a_* = −3.2) and Arg-58′ (Δp*K_a_* = −5.5). Binding of AVG to wild type CBL increases the p*K_a_* of Tyr-111 by over 2 units, although the p*K_a_* of Arg-58′ remains unchanged. In contrast, the binding of l-Ala-P to CBL(P113S) does not appear to alter the p*K_a_* of Tyr-111, provided Ser-113 is oriented toward it. When Ser-113 is oriented toward solvent instead, the p*K_a_* of Tyr-111 increases 3 units on binding the inhibitor. In their inhibitor-bound external aldimine forms, with Ser-113 oriented toward the active site, the difference in theoretical Tyr-111 p*K_a_* values between CBL and CBL(P113S) is estimated to be 5.6 units.

**TABLE 4:** **Selected side chain p*K_a_* values in liganded and unliganded CBL variants, as estimated by PROPKA 3.1**

Enzyme and ligand (Ser-113 rotamer)	p*K_a_* of Tyr-111	p*K_a_* of Arg-58`	PROPKA input (PDB code)
CBL	14.7	22.4	1CL1
CBL(P113S)	11.5	16.9	4ITG
CBL; AVG	17.0	22.5	1CL2
CBL(P113S); l-Ala-P (Ser-113 to Tyr-111)	11.4	19.8	4ITX
CBL(P113S); l-Ala-P (Ser-113 to solvent)	14.6	19.9	4ITX

## Discussion

### 

#### 

##### Reciprocally Promiscuous but Non-homologous Enzymes

The prevailing model for enzyme evolution invokes primordial cells containing a small number of genes, with each of these genes encoding a multifunctional protein (41). From this rudimentary starting point, gene duplication and divergence could give rise to the highly active, highly specific enzymes that are hallmarks of modern metabolic pathways. In many cases, the promiscuous activities of modern specialist enzymes are assumed to be evolutionary vestiges of their ancient multifunctional ancestors. Indeed, patterns of shared and overlapping promiscuous activities are common among the homologous members of enzyme superfamilies (42–44).

To our knowledge, this study reports the first example of reciprocal promiscuity between non-homologous enzymes. Instead of sequence similarity or a shared fold, ALR and CBL each utilize the powerful catalytic cofactor PLP.

##### ALR Is a Promiscuous and Evolvable CBL

The PLP-mediated cystathionine β-elimination activity of ALR was sufficient for enabling the slow growth of *E. coli* Δ*metC* cells. A single round of directed evolution yielded ALR(Y274F), which was amenable both to kinetic characterization and to structure determination. The conservative Y274F mutation enlarged the active site pocket just enough that cystathionine could enter it and form an external aldimine with PLP (Fig. 2).

Although ALR(Y274F) is able to bind cystathionine productively, its turnover number for β-elimination is very low (*k*_cat_ = 0.0028 s^−1^ ≈10 h^−1^). In CBL, Lys-210 mediates proton transfer from Cα to Sγ, with the elimination of homocysteine being rate-determining (36). In ALR, Tyr-255′ deprotonates l-Ala during the racemization reaction, and our docking analysis (Fig. 3*A*) shows that it is similarly well placed to deprotonate l-cystathionine. However, it is less clear whether Tyr-255′ is able to reorient to transfer this proton to the homocysteine leaving group (Fig. 3*B*). Furthermore, and in contrast to most PLP-dependent enzymes, ALR deprotonates the cofactor by placing an arginine (Arg-209 in the *E. coli* enzyme) adjacent to the nitrogen of the pyridine ring (4). This results in the formation of an unusually unstable carbanionic quinonoid intermediate during alanine racemization (45). In turn, this favors rapid reprotonation of Cα (*i.e.* racemization) by either Lys-34 or Tyr-255′, instead of the (slower) reorientation of these residues to protonate C4′ of the cofactor, which would lead to transamination (4). In CBL, the presence of Asp-185 ensures that the pyridine nitrogen is protonated (11), the quinonoid is stabilized, and Lys-210 can reorient to mediate proton transfer. In combination, the short-lived carbanionic intermediate and inefficient proton transfer to Sγ appear to ensure that ALR(Y274F) is a rudimentary CBL, at best.

##### CBL Is a Promiscuous and Evolvable ALR

Having characterized the promiscuous activity of ALR, we were surprised to discover that *E. coli* CBL is also a promiscuous ALR. As with ALR, the promiscuous activity of CBL could also be readily improved in a single round of epPCR and selection. The promiscuous activity of ALR(Y274F) was characterized by a good Michaelis constant but poor turnover. In contrast, the promiscuous activities of CBL and its Pro-113 mutants were characterized by relatively poor Michaelis constants but good turnover numbers (Table 2). Most notably, the CBL(P113S) variant had a *k*_cat_ for l-Ala racemization that was 22 s^−1^ per active site. This is comparable with the turnover number of ALR for the same reaction (*k*_cat_ = 55 s^−1^ per active site).

**FIGURE 5.**
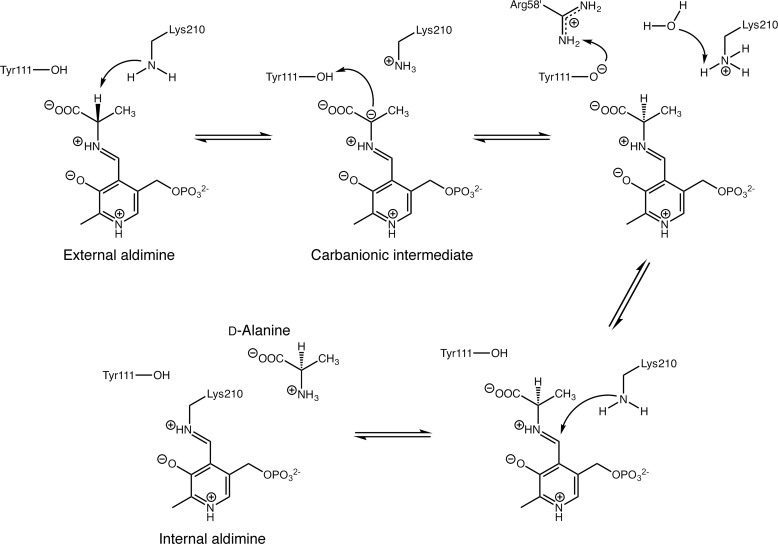
**Reaction scheme for racemization of l-alanine, catalyzed by CBL(P113S).** Estimates from PROPKA suggest that Tyr-111 is likely to be protonated when the l-Ala-PLP external aldimine is formed. When CBL catalyzes cystathionine β-elimination, the carbanionic intermediate is stabilized as the ketamine quinonoid (36). Here, we show instead the resonance structure of the carbanionic intermediate that is consistent with the ALR mechanism (22). Also, as done previously (22), we have shown the proton exchanges at Tyr-111 (involving Arg-58′) and Lys-210 occurring from the same intermediate, but this is not a necessity. Reverse transaldimination to regenerate the Lys-210-PLP Schiff base is assumed to occur as described for the cystathionine β-elimination reaction.

In the CBL active site, Lys-210 is ideally positioned to act as the catalytic base that deprotonates l-Ala after formation of the external aldimine (Fig. 5). It plays the same role in deprotonating the native substrate, l-cystathionine (36). Furthermore, Lys-210 is tethered in place by hydrogen bonds with the side chain hydroxyl groups of Tyr-56′ and Ser-339. This constrains the reaction specificity of CBL, by disfavoring protonation at the C4′ position of the cofactor (39). As discussed above, ALR achieves the same outcome by destabilizing the quinonoid intermediate. Each enzyme has evolved to minimize competing transamination reactions, coincidentally without narrowing its reaction specificity with respect to racemization and β-elimination.

A second parallel between the two active sites concerns the roles of Tyr-56′ in CBL and Tyr-343 in ALR. Both tyrosines help to anchor the cofactor phosphate, via hydrogen bonds of 2.7 and 2.6 Å, respectively. Each is also oriented so that, during the racemization reaction, the side chain of the substrate must move toward and past it to adopt the planar carbanionic intermediate and then the racemized product. In *G. stearothermophilus* ALR, mutation of the equivalent tyrosine (Tyr-354) to Asn yielded a bi-functional alanine/serine racemase (46). It remains to be tested whether a comparable mutation could broaden the substrate range of CBL acting as a racemase.

Completing the alanine racemization reaction requires protonation of the carbanionic intermediate from the opposite face than Lys-210. The structural data and our analysis of the CBL(Y111F) variant imply that Tyr-111 is the catalytic acid for racemization of l- to d-alanine (Fig. 5). An equivalent mutation (Y265F) has been made in the *G. stearothermophilus* ALR (19). In this enzyme, Tyr-265′ (Tyr-255′ in *E. coli* ALR) is the catalytic acid for the racemization of d- to l-alanine. We observed a 6,000-fold decrease in l-Ala racemization activity (from a low starting point; *k*_cat_/*K_m_* = 65 s^−1^·m^−1^) for CBL(Y111F), whereas the d-Ala racemization activity of *G. stearothermophilus* ALR(Y265F) was decreased by 60,000-fold (19).

In our l-Ala-P-PLP structure (Fig. 4*D*), the distance from the exocyclic oxygen atom of Tyr-111 to Cα of the substrate analogue is 4.2 Å. Attaining the planar intermediate will bring Cα closer to the catalytic acid, albeit in an orientation that is still not optimal for proton transfer. Nevertheless, the equivalent distance in our l-Ala-P-PLP structure of ALR(Y274F), between Cα and the catalytic acid Lys-34, is almost identical at 4.1 Å.

Previous structural and mutagenesis studies have interrogated the role of Tyr-111 in the native activity of CBL. Early structural work suggested that it might act as a catalytic acid/base, abstracting a proton from the α-amino group of cystathionine during transaldimination and then donating it to the aminoacrylate that is formed after homocysteine has been eliminated (11). This model required the nearby side chain of Arg-58′ to lower the p*K_a_* of the Tyr-111 side chain to the extent that it was a phenolate ion when substrate entered the active site. However, detailed kinetic analyses have ruled out a role for Tyr-111 in proton transfer (39). Our data on the alanine racemase activities of CBL and CBL(P113S) are consistent with the latter study. Our estimate of the Tyr-111 side chain p*K_a_* (14.7; Table 4) suggests that it will be uncharged in the substrate-free internal aldimine form of the enzyme. As predicted previously (11), this p*K_a_* increases when the substrate analogue, AVG, enters and increases the hydrophobicity of the active site. However, PROPKA 3.1 estimates that the presence of serine at position 113 will lower the theoretical p*K_a_* of Tyr-111 to ∼11.5, with or without bound substrate. This is likely due to the new hydrogen bond between Ser-113 and Arg-58′ (Fig. 4*C*) lowering the p*K_a_* of the latter and establishing a proton relay. The implication is that Tyr-111 is protonated when l-Ala (or l-Ala-P) binds but is better able to donate that proton during racemization (Fig. 5). By sampling two side chain conformations (Fig. 4*D*), Ser-113 also provides a route to shuttle a proton from bulk solvent back to Tyr-111, via Arg-58′, to complete the catalytic cycle (Fig. 5). Directed evolution acted to optimize the side chain p*K_a_* of a key catalytic residue. This was a less intuitive outcome than our initial prediction (*i.e.* repositioning of Tyr-111 to effect protonation of the carbanionic intermediate), and it highlights the ability of directed evolution to yield mechanistically unexpected solutions to challenges in enzyme engineering and evolution.

The native activity of CBL was reduced in all four of the Pro-113 variants that we characterized, by 3–16-fold (Table 2). In each mutant, most of this reduction in *k*_cat_/*K_m_* resulted from an increase in the *K_m_* value for cystathionine. One possible explanation involves Arg-58′, which plays an additional role by interacting with the distal carboxylate of cystathionine (47). Compared with proline, the side chains that we introduced at position 113 are each likely to partially occlude the space for this interaction, and/or alter the bonding capability of Arg-58′ (such as by introducing a new hydrogen bond in the case of Ser-113; Fig. 4*C*). Further structural studies with cystathionine analogues, such as AVG or the bis-hydrazino molecules described recently (48), will be useful for clarifying the effects on CBL activity of mutating Pro-113.

##### Evolutionary Considerations

PLP-dependent enzymes have attracted considerable interest as exemplars of protein evolution, due to the impressive diversity of their folds and functions. A parsimonious model suggests that the initial step in the evolution of each fold type was to constrain the reactivity of PLP to an overall reaction type, with narrowed substrate specificities arising later (49). This model would predict, for example, that all cystathionine β-lyases belong to fold type I and all alanine racemases belong to fold type III. However, our study adds to the body of evidence that convergent evolution has been common (2, 49, 50) and that there can be very short mutational trajectories between PLP-dependent enzymes with different reaction specificities.

Enzymes that catalyze β-elimination reactions and adopt fold types I, II, and IV have been characterized (2). It has been noted that this example of convergent evolution is unsurprising, because β-elimination of a good leaving group is highly facile and may simply require mutations that improve the binding of substrates with β leaving groups (50). Our discovery that ALR is a promiscuous CBL adds fold type III to the list of scaffolds that can support β-elimination reactions. As predicted (50), the first step in evolving a more efficient enzyme, the Y274F mutation, was to improve substrate binding. Further evolutionary enhancement of catalytic efficiency is likely to involve more wholesale changes to the active site. An ALR-based scaffold with native levels of CBL activity is likely to require the R209E mutation (to protonate the pyridine ring of the cofactor) and perhaps the mutation of Tyr-255′ to Lys to introduce the conformational flexibility required for effecting efficient proton transfer from Cα to Sγ. It is noteworthy that the former mutation improved the transamination activity of *G. stearothermophilus* ALR by 5.4-fold (32).

Although the *in vitro* kinetic parameters of ALR(Y274F) are poor, it is important to note that selection acts to improve organismal fitness (and not necessarily *k*_cat_ or *K_m_*). The intracellular performance of ALR(Y274F) is substantially better than its kinetics suggest. Although its *k*_cat_/*K_m_* value for the CBL reaction is 2.7 × 10^6^-fold lower than CBL itself, cells expressing ALR(Y274F) formed regular colonies in <48 h (∼2-fold slower than the same strain expressing CBL). One factor contributing to this apparent discrepancy between *in vitro* activity and *in vivo* performance is likely to be the high enzyme concentrations found *in vivo*. The conditions for inducing ALR(Y274F) expression were relatively mild (5 μm IPTG). Nevertheless, the volume of the *E. coli* cytoplasm is very small (∼1.25 fl), and in a previous study we estimated that the concentration of the rescuing enzyme may approach the millimolar range (26). We recently catalogued a number of comparable examples from other groups, in which enzymes have been replaced by weakly active alternatives, but cell growth has barely been diminished (51).

Conversely, another implication may be that CBL is “better than it needs to be,” *i.e.* mutations that erode its kinetic parameters for cystathionine β-elimination are unlikely to affect growth of the host cell. There is mounting evidence that many enzymes share this feature, presumably reflecting past evolutionary pressures (51).

The promiscuous activity of CBL was significantly greater than that of ALR and confirms that fold type I is compatible with racemase activity. Indeed, the alanine racemase from the fungus *Tolypocladium inflatum*, which provides the d-Ala required for cyclosporin A biosynthesis, is predicted to adopt this fold (52). With a *k*_cat_ of 3.8 s^−1^ and a *k*_cat_/*K_m_* of 5.4 × 10^2^ s^−1^·m^−1^, the *T. inflatum* enzyme has similar ALR activity to CBL(P113S) (Table 2).

CBL(P113S) has a *k*_cat_/*K_m_* value for l-Ala racemization that is only 170-fold lower than ALR itself. Thus, it mimics a bi-functional ancestor of the sort envisaged by Jensen (41). Furthermore, genome reduction is a pervasive mode of bacterial evolution (53). The evolutionary emergence of a bi-functional enzyme such as CBL(P113S) could remove any need to retain a specialized ALR and allow a bacterium to streamline its genome by losing its *alr* gene. A comparable event has occurred in the evolution of *Chlamydia trachomatis*, which has a small genome (1.0 Mbp) and uses a bi-functional TrpF enzyme to catalyze reactions in the tryptophan and tetrahydrofolate biosynthetic pathways (54). Another bacterium with a small genome (2.8 Mbp), *Treponema denticola*, uses a homologue of CBL, cystalysin, to catalyze cleavage of l-cysteine to pyruvate, NH_3_, and H_2_S. This enzyme has promiscuous ALR activity (55), although *T. denticola* also contains a specialist alanine racemase (with NCBI accession number NP_971703). One might predict that *T. denticola* will evolve to lose the gene for this racemase in future. This appears to have occurred already in *Wolbachia* strains (with genomes of ∼1.1 Mbp), in which it has been reported that CBL has ALR activity (albeit not quantified) (56). Although *Wolbachia* strains lack a specialist alanine racemase, they are also methionine auxotrophs (57), so CBL may be maintained solely for its ALR functionality in these organisms.

##### Concluding Remarks

We have demonstrated that two non-homologous specialized enzymes from *E. coli* are as little as one mutation away from being physiologically relevant replacements for each other. This emphasizes the catalytic versatility of PLP and the ease with which new functions can evolve on different PLP-binding protein scaffolds.

With the cofactor driving catalysis, there appears to be nothing that inherently constrains one function to one fold among the PLP-dependent enzymes. Given the preponderance of cofactor-mediated promiscuity, the short mutational routes between functions, historical contingency, and the sheer depth of evolutionary time, this implies that most (if not all) PLP-dependent functions will ultimately be discovered on most (if not all) types of PLP-binding fold. It will be (and already will have been) difficult to assign physiological functions correctly to PLP-binding enzyme from genome data alone. Our data also demonstrate that overlapping patterns of catalytic promiscuity do not necessarily imply homology in PLP-dependent enzymes. Thus, the evolutionary processes that have given rise to this diverse group of enzymes are much richer than those encapsulated by simple models that invoke tidy progressions from multifunctional ancestors to specialist descendants.

## Experimental Procedures

### 

#### 

##### Materials

Molecular biology enzymes were from New England Biolabs (Ipswich, MA). Oligonucleotides were from Integrated DNA Technologies (Coralville, IA). Chemicals were from Sigma, unless noted otherwise. IPTG was from Gold Biotechnology (St. Louis, MO). l-Cystathionine was dissolved in 0.04 n HCl to make 25 mm stocks. PLP was dissolved in 1 n HCl to make 100 mm stocks. DNA sequencing was done by Macrogen (South Korea).

##### Bacterial Strains and Media

The *E. coli* Δ*metC* and Δ*metE* strains were from the Keio collection (58). Under non-selective conditions, these strains were maintained in LB. *E. coli* strain MB2795 (Δ*alr* Δ*dadX*) was grown in the presence of 0.5 mm d-Ala under non-selective conditions. All plasmids were maintained using appropriate antibiotics as follows: carbenicillin, 50 μg·ml^−1^; chloramphenicol, 34 μg·ml^−1^; or kanamycin, 30 μg·ml^−1^. M9/glucose was used as the selective growth medium for the *E. coli* Δ*metC* and Δ*metE* strains. M9 plates contained 1 × M9 salts, 2 mm MgSO_4_, 0.1 mm CaCl_2_, 0.4% (w/v) glucose, and 1.5% (w/v) agar. For prolonged incubations (>2 days), all agar plates were placed in airtight plastic containers lined with moistened paper towels to prevent overdrying of the agar.

##### Selection of Suppressors

The pooled plasmids of the ASKA ORF library (23) were used to transform *E. coli* MB2795 by electroporation. Recovered cells were washed three times with 1× M9 salts before an aliquot containing ∼36,000 transformed cells (estimated by plating dilutions on non-selective medium) was spread on LB-chloramphenicol supplemented with 50 μm IPTG, but in the absence of d-Ala. The plate was incubated at 30 °C for 7 days, and colonies that formed were used as the source of template DNA for PCR, using the backbone-specific primers pCA24N.for and pCA24N.rev (10). The resulting PCR products were sequenced to reveal the identity of the ORFs.

##### Retransformation Tests

After each rescuing ORF had been identified, the plasmid from the corresponding ASKA clone was isolated. Aliquots of *E. coli* MB2795 were transformed with each purified plasmid. After recovering the cells in SOC medium supplemented with d-Ala at 37 °C for 1 h, the cells were washed with 1× M9 medium. A total of 250–1,000 transformed cells were spread on LB-chloramphenicol plates containing 50 μm IPTG. Each agar plate was incubated at 30 °C for at least 7 days.

##### Growth Complementation Assays

For complementation of the *E. coli* Δ*metC* and Δ*metE* strains, an overnight culture of each test clone was centrifuged at 12,000 × *g* for 4 min. The cell pellet was washed three times with 1 ml of 1 × M9 medium. The final cell resuspension was diluted to an *A*_600_ of 0.1 using 1× M9 medium. A 2.5-μl aliquot was spread on M9/glucose plates containing 50 μm IPTG. Cell counts were obtained by plating appropriate dilutions of each culture on LB agar plates. The M9/glucose plates were incubated at 28 °C for at least 7 days, whereas the LB agar plates were incubated overnight at 37 °C. Complementation tests with *E. coli* MB2795 were carried out the same way, except that LB was used to wash the cell pellet, growth tests were on LB agar plates with IPTG (50 μm), and total cell counts were obtained by spreading dilutions of the cell resuspension on LB-chloramphenicol plates with 0.5 mm d-Ala.

##### Enzyme Expression and Purification

All enzyme variants were expressed with His_6_ tags at their N termini, from the respective ASKA plasmids (with their C-terminal GFP tags removed). A 4-ml culture of *E. coli* cells harboring each expression vector was grown to saturation, before being used to inoculate 250 ml of LB broth containing appropriate antibiotics. At *A*_600_ ∼0.5, IPTG (0.5 mm, final concentration) was added to the culture. Protein overexpression was carried out at 28 °C for another 5–16 h. Cells were then collected by centrifuging at 5,000 × *g* and 4 °C for 20 min.

Column Buffer (CB) consisted of 50 mm potassium phosphate buffer (pH 8.0), 300 mm NaCl, 3 mm imidazole, 1 mm β-mercaptoethanol, 10% (v/v) glycerol, and 10 μm PLP. Each cell pellet was resuspended with 4 ml of this buffer/g (wet pellet weight). Lysozyme (0.5 mg·ml^−1^), DNase I (2 units), and protease inhibitor mixture (50 μl) were added, and the suspension was incubated on ice for 45 min. Cell lysis was by 10 cycles of sonication (amplitude 45%; ultrasonic processor S-4000, Misonix) on ice. The lysate was clarified by centrifugation at 20,000 ×*g* for 35 min at 4 °C. The soluble fraction was incubated with 1 ml of Talon® metal affinity resin (Clontech) at 4 °C for 2 h. Next, the resin was washed four times with 3 ml of CB, and finally, one time with 3 ml of CB that contained 15 mm imidazole. The His_6_-tagged enzyme was eluted with 3 ml of Elution Buffer (50 mm potassium phosphate buffer (pH 8.0), 300 mm NaCl, 150 mm imidazole, 1 mm β-mercaptoethanol, 10% (v/v) glycerol). All enzyme variants were judged to be >95% pure by SDS-PAGE analysis.

Purified enzymes were dialyzed extensively against Dialysis Buffer (50 mm potassium phosphate (pH 8.0), 150 mm NaCl, 10% (v/v) glycerol) at 4 °C before being concentrated using a centrifugal filtration concentrator (GE Healthcare, Little Chalfont, UK). Aliquots of each purified enzyme were stored at either 4 or −80 °C. Total enzyme concentrations were quantified using molar extinction coefficients that were calculated as described previously (59). The concentrations of PLP-bound enzymes were determined using a molar extinction coefficient of 8,450 m^−1^·cm^−1^ for PLP (60). In all batches of purified ALR, ALR(Y274F), and CBL, at least 70% of the total protein was PLP-bound and hence catalytically active. For the CBL variants, the proportions of PLP-bound protein were 30–50%. All enzymes were used within 2 weeks of being purified.

##### Steady State Kinetic Assays

Activity assays were performed using a Cary 100 UV-visible spectrophotometer (Agilent Technologies; Santa Clara, CA). Reactions were measured in quartz cuvettes with a path length of 1 cm.

A continuous coupled assay, based on that described previously (61), was used for detecting the racemization of l-Ala to d-Ala. The 1-ml reaction mixtures contained 100 mm CHES (pH 9.0), 0.2 mm NADH, 1 unit of d-amino acid oxidase, and 120 units of lactate dehydrogenase, with the l-Ala concentration varied. Each mixture was pre-incubated at 37 °C for 2 min, and then enzyme was added to initiate the reaction. The final enzyme concentrations in each reaction were as follows: ALR, 8.4 nm; and CBL variants, 29–158 nm. The oxidation of NADH to NAD^+^ during the reaction was monitored by a decrease in *A*_340_, and reaction rates were quantified using a molar extinction coefficient of 6,220 m^−1^·cm^−1^ for NADH. The substrate concentrations used for ALR-catalyzed assays ranged from (0.3–10) × *K_m_*, whereas those catalyzed by the CBL variants ranged from ≤0.5 to ≥2 × *K_m_*. In the absence of enzyme, we found that the average rate of spontaneous NADH oxidation was <0.5 μm·min^−1^ (which was <2% of the maximal rate of ALR-catalyzed NAD^+^ formation). This background rate was measured and subtracted from each assay.

The continuous assay for cystathionine β-elimination was modified from Uren's protocol (62). Each reaction contained 50 mm Tris·HCl (pH 8.8) (or 100 mm Tris·HCl (pH 8.8), for assays with ALR variants), 0.4 mm DTNB, and enzyme. The final enzyme concentrations used in each reaction were as follows: CBL, 11 nm (in 1-ml assays); evolved CBL variants, 6.5–20.2 nm (in 1-ml assays); and ALR variants, 0.8–7 μm (in 0.5-ml assays). Each reaction was pre-incubated at 37 °C for 2 min, and l-cystathionine was added to initiate the reaction. The thiol group of homocysteine (produced from the enzyme-catalyzed reaction) reacted with DTNB to form the 2-nitro-5-thiobenzoate dianion. The formation of 2-nitro-5-thiobenzoate dianion was monitored by an increase of *A*_412_ and was quantified using its molar extinction coefficient of 14,150 m^−1^·cm^−1^ (63). For CBL assays, the substrate concentrations typically ranged from (0.25–3) × *K_m_*, whereas those containing the evolved CBL variants ranged from 0.1 to ≥1.6 × *K_m_*. Assays with the ALR(Y274F) variant contained substrate concentrations ranging from (0.6–3) × *K_m_*. The background rate of spontaneous DTNB decomposition was measured and subtracted from each assay.

Initial rate data were fitted to the Michaelis-Menten equation using Origin software (OriginLab; Northampton, MA). All Michaelis-Menten plots were constructed from activity data obtained at 7–9 substrate concentrations. The concentration of active enzyme in each assay was assumed to be the concentration of PLP-bound enzyme.

##### Random Mutagenesis of alr

Random mutations were introduced into the *alr* gene using the GeneMorph II Random Mutagenesis kit from Agilent (La Jolla, CA). The epPCR had a total volume of 100 μl and contained 1× Mutazyme buffer, 2.5 units of Mutazyme DNA polymerase, 0.8 mm dNTPs, 0.5 μm primer pCA24N.for, 0.5 μm primer ALR.rev (5′-GCG GCC GCA TAG GCC TTA ATC CAC GTA TTT C-3′), and 570 ng of plasmid pCA24N-*alr* (corresponding to 100 ng of the *alr* template). The thermocycling conditions were 95 °C for 2 min, 35 cycles of 95 °C (30 s), 58 °C (30 s), 72 °C (70 s), and one final extension step at 72 °C for 2 min. The 1.2-kb amplified product was purified using a QIAquick spin column (Qiagen, Valencia, CA) and cloned into the SfiI sites of the pCA24N vector (23). *E. coli* Δ*metC* cells were transformed with the ligated products by electroporation. Transformed cells were plated on LB-kanamycin-chloramphenicol library plates (245 × 245 mm, Corning) and allowed to grow at 37 °C for 15 h. The *alr* inserts from 20 randomly selected colonies were sequenced using primers pCA24N.for and pCA24N.rev2 (5′-CAA ATC CAG ATG GAG TTC TGA GG-3′) to estimate the mutation spectrum of the library.

##### Selection for ALR Variants with Higher CBL Activity

Colonies harboring the randomly mutated *alr* variants were pooled by scraping them off the library plates in aliquots of LB medium, and then a 10-μl aliquot of the pool was used to inoculate 50 ml of fresh LB/kanamycin/chloramphenicol medium. At *A*_600_ ∼0.8, the cells were harvested by centrifugation at 2,200 × *g* and 4 °C for 15 min, followed by a wash with 50 ml of 1 × M9 medium. The cells were spread on an M9/glucose library plate supplemented with 5 μm IPTG. For side-by-side comparison, *E. coli* Δ*metC* cells carrying pCA24N-*alr* were spread on an additional plate. The same *E. coli* strain harboring an empty ASKA vector (pCA24N-NoIns (24)) was used as a negative control, whereas cells harboring pCA24N-*metC* were used as a positive control. After 2 days of incubation at 28 °C, a random subset of the colonies that had appeared on the library plate were picked. The randomly mutated *alr* allele from each colony was amplified by PCR with primers pCA24N.for and pCA24N.rev2, and the products were sequenced.

##### Random Mutagenesis of metC

The *metC* gene was amplified in an epPCR, as described above for *alr* except that the primers used were pCA24N.for and metC(GFP-).rev (5′-GCG GCC GCA TAG GCC TTA TAC AAT TCG CGC A-3′), and the template was 1.3 μg of plasmid pCA24N-*metC* (corresponding to 300 ng of the *metC* gene). The thermocycling conditions were 95 °C for 90 s, 30 cycles of 95 °C (30 s), 54 °C (30 s), 72 °C (90 s), and a final cycle of 72 °C for 60 s. The 1.2-kb product was digested with SfiI and ligated with the pCA24N(KanR) vector (a derivative of pCA24N, in which the chloramphenicol resistance marker had been replaced by a kanamycin marker), after it had been digested with the same restriction enzyme. The ligated products were used to transform *E. coli* DH5α-E (Invitrogen) by electroporation, and transformed cells were grown on LB-kanamycin plates at 37 °C for 16 h. Fourteen colonies were picked at random for DNA sequencing to estimate the mutation spectrum of the library. The remainder of the transformed cells (∼1.5 × 10^5^ colonies) were pooled, and the plasmids were isolated. The plasmid pool was used to transform *E. coli* MB2795. Transformed cells were spread on a library plate of LB-kanamycin supplemented with 0.5 mm d-Ala. Approximately 3 × 10^7^ colonies formed after 16 h of incubation at 37 °C. This ensured that 100% of the initial library was sampled (64).

##### Selection for CBL Variants with Higher ALR Activity

The *E. coli* MB2795 colonies carrying randomly mutated *metC* variants were pooled, and a 10-μl aliquot was transferred into 50 ml of fresh LB-kanamycin that had been supplemented with d-Ala (0.5 mm). The cells were propagated at 37 °C to an *A*_600_ of 0.4. The culture was diluted appropriately, so that an estimated 1.0 × 10^6^ colony-forming units were spread on a library plate of LB-kanamycin supplemented with 5 μm IPTG. After incubating the plate at 28 °C for 42 h, 48 random colonies were restreaked on fresh selective plates, both with and without 5 μm IPTG, to confirm the growth phenotype of the clones. Clones with reproducible phenotypes were subjected to PCR screens and DNA sequencing analysis.

##### Crystallization, Data Collection, and Structure Solution of ALR(Y274F) and CBL(P113S)

Immediately prior to crystallization attempts with ALR(Y274F), a 20-ml spin concentrator with nominal molecular mass cutoff of 50 kDa (GE Healthcare) was used to exchange the protein into a buffer consisting of 20 mm Tris·HCl (pH 8.0) and 100 μm PLP. Protein was crystallized by vapor diffusion using the hanging drop method at a temperature of 291 K and with a reservoir solution (500 μl) consisting of 1.6 m ammonium sulfate, 100 mm HEPES (pH 7.0). A 2:1 ratio of protein to crystallization buffer yielded yellow-colored hexagonal plate crystals that appeared overnight and grew to approximate dimensions of 0.4 × 0.4 × 0.1 mm over 7 days. After a week, crystals were supplemented with 10 mm PLP for 3 days to ensure complete cofactor incorporation.

Prior to data collection, an inhibited ALR(Y274F) structure was produced by soaking pre-formed crystals with 10 mm l-Ala-P. Inhibitor soaking was for 30 min, before a brief soak in crystallization buffer supplemented with 30% glycerol, and then flash-cooling in liquid nitrogen. Data were collected to 2.25 Å for the inhibitor-treated crystal and to 1.86 Å for an untreated crystal on a Rigaku MicroMax-007 HR rotating anode instrument equipped with a MAResearch Mar345dtb detector and Oxford Cryosystem Cobra cooling system. Data were processed using XDS (65) and scaled using SCALA (66). The structures were solved by molecular replacement using the wild type *E. coli* ALR structure (PDB 2RJG) and the program PHASER (67) and were modeled using COOT (68). The structures were refined using REFMAC5 (69) to *R*-factors of 17.5% (uninhibited) and 16.7% (inhibited), and free *R* values of 20.4 and 20.2%, respectively. The apparent space group of these crystals was *P*622, but subsequently the true space group was determined as *P*6, with twin refinement in REFMAC5 improving the refinement statistics markedly. The final models were assessed by Ramachandran plot and geometrical analysis using both the MOLPROBITY server (70) and the PDB validation server. PLP and l-Ala-P-PLP aldimine molecules were modeled into discrete and unambiguous electron density, and sulfate and water molecules were added in the final rounds of refinement. The non-inhibited structure contains four protein molecules (two dimers), four PLPs, five sulfates, three glycerols, and 467 water molecules. The inhibited structure contains four protein molecules, three l-Ala-P-PLP aldimines, eight sulfates, and 236 water molecules. Active site electron density is shown in the supplemental Fig. S1, and data collection and refinement statistics are in supplemental Table S2.

The CBL(P113S) protein was treated similarly to ALR(Y274F), apart from the following specific details. Buffer exchange prior to crystallization used 5 mm HEPES (pH 7.5) supplemented with 10 μm PLP. Protein was finally concentrated to 7.5 mg·ml^−1^ and crystallized by vapor diffusion using the hanging drop method and with a reservoir solution consisting of 10% PEG400, 100 mm HEPES (pH 7.3), and 150 mm CaCl_2_. Various ratios of protein to reservoir solution were mixed (1 + 1 μl, 2 + 2 μl, 3 + 1.5 μl, and 1.5 + 3 μl) and inverted over the reservoir solution. In all conditions, yellow-colored coffin-shaped crystals grew overnight, with the largest reaching a final size of ∼0.2 × 0.2 × 0.4 mm within 2 days. A large and perfect looking crystal was coated in a 70:30 mixture of paratone N and mineral oil before flash-cooling in liquid nitrogen; it was diffracted to a resolution of 1.74 Å. The structure was solved by molecular replacement using the wild type CBL structure (PDB 1CL1). The P113S mutation was incorporated, and a HEPES molecule plus 379 water molecules were included in the final model. The PLP cofactor was clearly observed in the structure at full occupancy.

An inhibited CBL(P113S) structure was produced by soaking a pre-formed crystal overnight in a crystallization solution supplemented with 10 mm l-Ala-P. Data were collected in-house and processed, and the structure was solved and refined as outlined above. The l-Ala-P-PLP aldimine was clearly observed in discrete and unambiguous electron density and was included in final refinement along with 514 water molecules. Active site electron density is shown in supplemental Fig. S1, and data collection and refinement statistics for the uninhibited and inhibited CBL(P113S) structures are in supplemental Table S2.

##### Modeling a Cystathionine-PLP Adduct into the ALR(Y274F) Active Site

A cystathionine-PLP adduct was built using the ligand building module in COOT (68). This molecule was docked into our crystal structure model of ALR(Y274F); to prepare the protein model, all ligands and water molecules were removed and hydrogen atoms added. Docking used the program GOLD version 5.2 (CCDC); the GOLD wizard and default docking parameters were used to produce an initial command file that was subsequently edited to include a scaffold match constraint. The PLP fragment of the l-Ala-P-PLP molecule in our inhibited ALR(Y274F) crystal structure was used as the scaffold, essentially forcing the PLP of the cystathionine-PLP adduct to assume the inhibited crystal structure position. The cystathionine fragment was allowed to rotate freely. Atom typing was assigned for both the protein and cystathionine-PLP molecules by GOLD, and 10 docking solutions were produced for manual inspection.

## Author Contributions

W. M. P. conceived and coordinated the study. V. W. C. S. designed and performed all of the molecular biology, enzyme kinetics and directed evolution experiments. Y. Y. and C. J. S. designed and conducted the crystallographic experiments. C. J. S. carried out the docking analysis. V. W. C. S., C. J. S., and W. M. P. analyzed the data and wrote the paper. All authors reviewed the results and approved the final version of the manuscript.

## Supplementary Material

Supplemental Data

## Footnotes

2 The abbreviations used are:

PLP: pyridoxal 5′-phosphate
ALR: alanine racemase
ASKA: A complete set of *E. coli* K-12 ORF archive
AVG: l-aminoethoxyvinylglycine
CBL: cystathionine β-lyase
DTNB: 5,5′-dithiobis(2-nitrobenzoic acid)
epPCR: error-prone PCR
IPTG: isopropyl β-d-1-thiogalactopyranoside
l-Ala-P: (*R*)-1-aminoethylphosphonic acid
PDB: Protein Data Bank
CHES: 2-(cyclohexylamino)ethanesulfonic acid
POOL: Partial Order Optimum Likelihood.
