# Supplementary material for: Mechanistic and Evolutionary Insights from the Reciprocal Promiscuity of Two Pyridoxal Phosphate-dependent Enzymes
Source: J Biol Chem. 2016 Jul 29;291(38):19873–87. doi: 10.1074/jbc.M116.739557 (PMC5025676; doi:10.1074/jbc.M116.739557)
Supplement: Supplemental Data [file supp_291_38_19873__index.html]

Mechanistic and Evolutionary Insights from the Reciprocal Promiscuity of Two Pyridoxal Phosphate-Dependent Enzymes — Mechanistic and Evolutionary Insights from the Reciprocal Promiscuity of Two Pyridoxal Phosphate-dependent Enzymes — Reciprocal Promiscuity in Two PLP-dependent Enzymes — Supplemental Data 

# Mechanistic and Evolutionary Insights from the Reciprocal Promiscuity of Two Pyridoxal Phosphate-dependent Enzymes

## Supplemental Data

- Supplemental Data (.pdf, 1.5 MB) - Supplemental Tables S1 and S2; Supplemental Figure S1.
