# Supplementary material for: Mechanistic and Evolutionary Insights from the Reciprocal Promiscuity of Two Pyridoxal Phosphate-dependent Enzymes
Source: J Biol Chem. 2016 Jul 29;291(38):19873–87. doi: 10.1074/jbc.M116.739557 (PMC5025676; doi:10.1074/jbc.M116.739557)

Mechanistic and Evolutionary Insights from the Reciprocal Promiscuity of Two Pyridoxal Phosphate-Dependent Enzymes

Valerie W. C. Soo, Yuliana Yosaatmadja, Christopher J. Squire, and Wayne M. Patrick

SUPPLEMENTAL TABLES

Table S1. Days to form colonies for various *E. coli* strains grown on M9/glucose medium, supplemented with IPTG (50 μM).

Over-expressed protein	Δ*metC*	Δ*metC* + L-methionine	Δ*metE*	Δ*metE* + L-methionine
CBL	1–2	1–2	NT*	NT*
None (empty vector)	No growth	1–2	No growth	2
ALR-GFP	5–6	NT*	NT*	NT*
ALR	3–4	1–2	No growth	2
Methionine synthase	NT*	NT*	2	2

*NT: Not tested.

Table S2. Crystal properties, data collection and refinement statistics.

	**ALR(Y274F)**	**ALR(Y274F) + L-Ala-P**	**CBL(P113S)**	**CBL(P113S) + L-Ala-P**
**PDB entry**	4WR3	4XBJ	4ITG	4ITX
**Data collection**				
Space group	*P*6	*P*6	$C222_1$	$C222_1$
Unit-cell parameters:				
a (Å)	148.08	147.98	59.93	59.97
b (Å)	148.08	147.98	152.77	153.22
c (Å)	163.69	163.58	150.85	150.91
α, β, γ (°)	90, 90, 120	90, 90, 120	90, 90, 90	90, 90, 90
Beamline	Rotating anode	Rotating anode	Rotating anode	Rotating anode
Resolution	1.86 (1.90-1.86)	2.25 (2.29-2.25)	1.74 (1.83-1.74)	1.61 (1.69-1.61)
Wavelength (Å)	1.5418	1.5418	1.5418	1.5418
$CC_{1/2}$	0.998 (0.632)	0.992 (0.617)	1.000 (0.976)	1.000 (0.881)
Completeness (%)	99.6 (93.8)	99.7 (97.0)	99.7 (98.3)	99.0 (93.7)
Observed reflections	168,143	95,615	71,753	89,828
$<I/\sigma(I)>$	18.5 (1.9)	10.7 (2.0)	35.7 (8.7)	25.6 (4.2)
Multiplicity	22.1 (16.4)	11.4 (10.2)	15.9 (14.8)	11.6 (9.7)
**Refinement**				
Resolution range	19.7-1.90	19.9-2.25	19.5-1.74	76.6-1.61
Reflections used	149,471	90,731	67,348	85,255
R factor	0.175	0.167	0.164	0.164
R_{free}	0.204	0.202	0.203	0.199
Average *B* factor (Å^2)	22.8	21.7	16.5	16.2
RMS deviation from ideal:				
Bond lengths (Å)	0.008	0.008	0.020	0.022
Bond angles (°)	1.349	1.142	2.126	2.157
Ramachandran plot (%):				
Favoured	96.8	96.2	97.2	97.8
Outliers	0.0	0.0	0.0	0.0
Molprobity server score	93rd percentile	96th percentile	93rd percentile	94th percentile

Data for the high resolution shell are shown in parentheses.

SUPPLEMENTAL FIGURE

Figure S1. 2Fo – Fc electron density maps of the PLP sites in ALR(Y274F), ALR(Y27F)–L-Ala-P, CBL(P113S) and CBL(P113S)–L-Ala-P. Each map is contoured at 1σ.

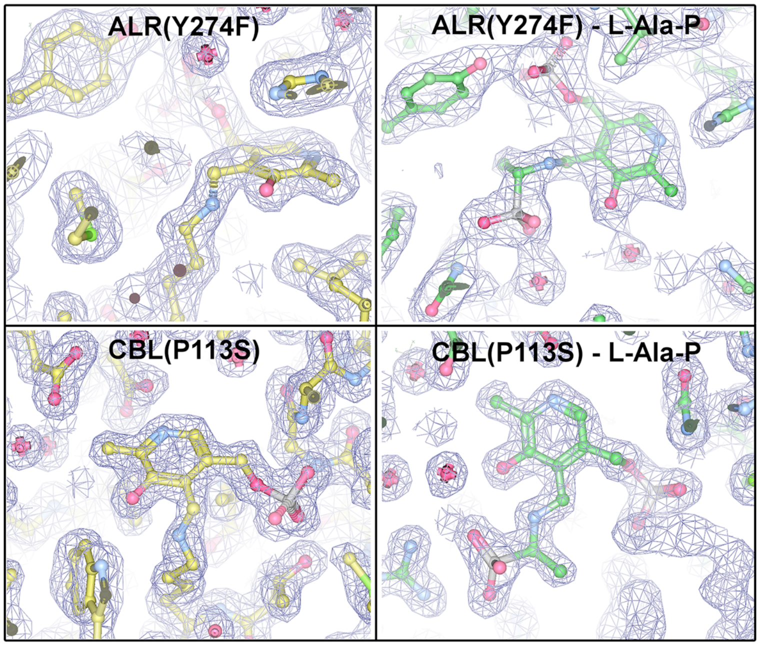

Supplement: Supplemental Data [file 10.1074_M116.739557_jbc.M116.739557-1.pdf]
